# circRNA ITGA7 restrains growth and enhances radiosensitivity by up-regulating SMAD4 in colorectal carcinoma

**DOI:** 10.1515/med-2022-0604

**Published:** 2023-01-11

**Authors:** Wei Li, Wancheng Wei, Dingyin Hu, Rutong Tang, Zikang Hu

**Affiliations:** Department of Anorectal Hemorrhoids, Gaozhou People’s Hospital, Gaozhou City, Guangdong Province, 525200 China; Department of Anorectal Hemorrhoids, Gaozhou People’s Hospital, No. 89, Xiguan Road, Gaozhou City, Guangdong Province, 525200 China

**Keywords:** circ_ITGA7, miR-766, SMAD4, tumorigenicity, radioresistance

## Abstract

Circular RNAs have been reported to be widely involved in cancer cell tumorigenesis and drug resistance; here, the aim of this study was to investigate whether circRNA Integrin Subunit Alpha 7 (ITGA7) (circ_ITGA7) was associated with the tumor growth and radiosensitivity of colorectal cancer (CRC). We found that circ_ITGA7 expression was lower in CRC tissues and cells than those in the normal tissues and cell lines according to quantitative real-time polymerase chain reaction. As shown by cell counting kit-8 assay, flow cytometry, colony formation assay, and xenograft experiment, ectopic overexpression of circ_ITGA7 remarkably restrained CRC tumor growth and enhanced radiosensitivity *in vitro* and *in vivo*. Mechanistically, circ_ITGA7 could target microRNA (miR)-766 to prevent the degradation of its target gene mothers against decapentaplegic homolog 4 (SMAD4), the binding between miR-766 and circ_ITGA7 or SMAD4 was first verified by dual-luciferase activity assay. Additionally, miR-766 up-regulation reversed the inhibitory effects of circ_ITGA7 on CRC growth and radiosensitivity. Besides that, inhibition of miR-766 reduced CRC cell growth and sensitized cells to radiotherapy, and these effects mediated by miR-766 inhibitor were rescued by the silencing of SMAD4. In all, circ_ITGA7 suppressed CRC growth and enhanced radiosensitivity by up-regulating SMAD4 through sequestering miR-766, providing an insight for the further development of CRC treatment.

## 1 Introduction

Colorectal cancer (CRC) is one of the most commonly diagnosed malignancies throughout the world. Attributing to the technological advances in early detection and intervention, the overall survival of CRC has partially improved; however, patients with advanced stage are difficult to completely eliminate with high frequency of metastasis and recurrence [1,2]. Radiotherapy (RT) is a commonly used nonsurgical modality for both curative and palliative therapies of many types of malignancies; moreover, RT alone or along with surgery is an important treatment option for CRC patients [3,4]. Unfortunately, the intrinsic and acquired radioresistance among a good deal of CRC patients limits the efficacy of RT [5,6]. Thus, further investigations on the molecular mechanisms of CRC tumor growth and radiosensitivity are helpful for improving CRC therapy.

Circular RNAs (circRNAs) are non-coding transcripts with a 3′,5′-phosphodiester bond at the junction site, they are widespread and abundant in eukaryotic transcriptome, and show high conservation across species [7]. Increasing studies have revealed that circRNAs play a significant role in numerous biological processes [8]. Importantly, dysregulation of circRNAs in many diseases including cancer has been observed [9,10], besides that, circRNAs are widely reported to involve in cancer cell tumorigenesis and drug resistance [11]. circRNA Integrin Subunit Alpha 7 (ITGA7) (circ_ITGA7, ID: hsa_circ_0026782) is derived from the ITGA7 gene and located at chr12:56094682–56094938. Li et al. showed that circ_ITGA7 was decreased in CRC; importantly, circ_ITGA7 overexpression repressed CRC growth and metastasis via blocking the Ras signaling pathway and up-regulating ITGA7 expression [12]. Besides, another study showed that circ_ITGA7 increased ASXL1 expression level by absorbing miR-3187-3p to reduce the proliferation rate of CRC cells [13]. Although these findings support the notion that abnormal expression of circ_ITGA7 is implicated in CRC tumorigenic molecular pathway, the function of circ_ITGA7 in radioresistance remains unclear.

Herein, the purpose of this study was to elucidate the expression profile of circ_ITGA7 in CRC, and investigate the action and mechanism of circ_ITGA7 in CRC tumor growth and RT sensitization.

## 2 Materials and methods

### 2.1 Clinical samples

Fifty-one pairs of CRC tissues and adjacent normal tissues were collected from patients who underwent surgical resection at Gaozhou People’s Hospital. All patients were newly diagnosed as CRC by pathological examination and received radiation therapy alone. All tissue samples were stored in −80°C until RNA extraction.


**Ethical approval and consent to participate:** The present study was approved by the ethical review committee of Gaozhou People’s Hospital. Written informed consent was obtained from all enrolled patients.
**Consent for publication:** Patients agree to participate in this work.

### 2.2 Cell culture

Human CRC cell lines HCT116 and DLD1, and normal colonic cell line FHC were provided by Cedarlane (Burlington, NC, USA). HCT116 and DLD1 cells were cultured in the Dulbecco’s Modified Eagle’s Medium (DMEM; Life Technologies, Scotland, UK) with 100 U/mL of penicillin and streptomycin (HyClone, Logan, UT, USA) and 10% fetal bovine serum (FBS; Hyclone). FHC cells were cultured in DMEM/F-12 (Life Technologies) plus 10% FBS, 10 ng/mL cholera toxin, 5 μg/mL insulin, 5 μg/mL transferrin, 100 ng/mL hydrocortisone, and 10 mM Hanks’ balanced salt solution. All cells were maintained in a humidified 5% CO_2_ incubator at 37°C.

### 2.3 Quantitative real-time PCR (qRT-PCR)

Nuclear and cytoplasmic separation was performed using RNA Subcellular Isolation Kit (Life Technologies) following the manufacturer’s instructions. For Actinomycin D treatment, cells were incubated with 2 μg/mL Actinomycin D or dimethylsulfoxide (as control) (Sigma-Aldrich, St. Louis, MO, USA) to block transcription at indicated time points. Total RNA from the tissues and cultured cells was prepared using RNeasy Mini Kit (Life Technologies). The single-stranded CDNA was synthesized in 25 µL reactions using SuperScriptIII reverse transcriptase (Takara Biotech, Otsu, Japan) with 2 µg of RNA. Then qRT-PCR was conducted using SYBR QPCR Mix (Toyobo, Tokyo, Japan). The level of glyceraldehyde-3-phosphate dehydrogenase (GAPDH) or U6 was simultaneously detected for normalization of circ_ITGA7, linear ITGA7 (Integrin Subunit Alpha 7) mRNA, miR-766, and SMAD4 (mothers against decapentaplegic homolog 4) mRNA expression employing the comparative Ct method. The primer sequences for qRT-PCR are:

circ_ITGA7: F 5′-GTGTGCACAGGTCCTTCCAA-3′, R 5′-TGGAAGTTCTGTGAGGGACG-3′;

ITGA7: F 5′-TATTGACTCGGGGAAAGGTCT-3′, R 5′-CCAGCCATCACTGTTGAGG-3′;

SMAD4: F 5′-CTCATGTGATCTATGCCCGTC-3′, R 5′-AGGTGATACAACTCGTTCGTAGT-3′;

miR-766: F 5′-CAATCCTTACTCCAGCCAC-3′, R 5′-GTGTCTTAAGGCTAGGCCTA-3′;

GAPDH: F 5′-GCACCGTCAAGGCTGAGAAC-3′, R 5′-TGGTGAAGACGCCAGTGGA-3′;

U6: F 5′-GCAGACCGTTCGTCAACCTA-3′, R 5′-AATTCTGTTTGCGGTGCGTC-3′.

### 2.4 Cell transfection

The pLC5-ciR-circ_ITGA7 overexpression plasmid (circ_ITGA7) and plasmid containing scrambled sequences (Vector), SMAD4-specific siRNA (si-SMAD4), and negative control siRNA (si-NC) were obtained from Genechem (Shanghai, China). Mature miR-766 mimics (miR-766), inhibitors (anti-miR-766), and the negative control miR (anti-NC or anti-miR-NC) were provided by GenePharma (Shanghai, China). Then transient transfections with 2 μg of circ_ITGA7, Vector, or 50 nM of si-SMAD4, si-NC, miR-766, anti-miR-766, or the negative control miR were performed using Lipofectamine^TM^ 3000 reagent (Thermo Fisher Scientific). Lentivirus particles encoding circ_ITGA7 (Lenti-circ_ITGA7) or Vector (Lenti-NC) were purchased from Genechem and then stably transfected into HCT116 cells at a multiplicity of infection of 25. After 2 weeks of screening with puromycin, stably expressed cells were selected for subsequent analysis.

### 2.5 Cell counting kit-8 (CCK-8) assay

Transfected HCT116 and DLD1 cells (1 × 10^4^/well) were seeded into 96-well plates, then cultured for 0, 1, 2, or 3 days before the addition 10 μL of CCK-8 (5 mg/mL) to the culture medium in each well. One hour after CCK-8 addition, cell proliferation was analyzed by reading the absorbances at 570 nm with a Viktor X3 reader (PerkinElmer, Turku, Finland).

### 2.6 Flow cytometry

For cell cycle analysis, transfected HCT116 and DLD1 cells were collected and fixed in 70% ethanol. After rinsing with phosphate-buffered saline (PBS), fixed cells were stained with propidium iodide (PI) (Life Technologies). Finally, cell cycle distribution was evaluated using a FACS Calibur flow cytometer (BD Biosciences, San Diego, CA, USA).

After assigned transfection, HCT116 and DLD1 cells were exposed to 0 or 6 Gy irradiation for 48 h. Then cells were harvested by trypsin, resuspended by 1× binding buffer and then stained orderly with fluorescein isothiocyanate-Annexin V (BD Biosciences) and PI (Life Technologies) for 15 min. Then cell apoptosis was determined using a BD Biosciences flow cytometer.

### 2.7 Colony formation assay

Transfected HCT116 and DLD1 cells were seeded into a six-well plate at 1 × 10^3^ cells per well and then irradiated with 0, 2, 4 or 6 Gy for 48 h. After incubation for 14 days at 37°C, cells were fixed with 4% paraformaldehyde (Sigma-Aldrich) for 30 min and then stained with 0.5% crystal violet (Sigma-Aldrich) for 15 min. Number of viable cells in five randomly selected fields were assayed. The survival fraction was calculated according to the formula: (number of colonies/number of cells plated)_irradiated_/(number of colonies/number of cells plated)_non-irradiated_.

### 2.8 Western blot

Cultured cells and tissues were disrupted by RIPA lysis and then quantified with the Bio-Rad protein assay kit (Bio-Rad, Richmond, CA, USA). Equivalent amounts of protein (50 µg per lane) were separated by 10% SDS-poly acrylamide gel electrophoresis and transferred to Clear Blot membrane-p (ATTO, Tokyo, Japan). Then primary antibody incubation and secondary antibody incubation were performed, membranes carrying protein blots were visualized by enhanced chemiluminescence detection system (Life Technologies). The antibodies used in this study included B-cell lymphoma-2 (Bcl-2; 1:1,000, ab194583), GAPDH (1:5,000, ab181602), Bcl-2-associated X protein (Bax; 1:1,000, ab32503), Vimentin (1:2,000, ab92547), E-cadherin (1:2,000, ab15148), and SMAD4 (1:5,000, ab40759) (Abcam, Cambridge, MA, USA).

### 2.9 Dual-luciferase activity assay

The pGL3-Basic Vector harboring the mutated (MUT) or wild-type (WT) miR-766 binding sequences in the 3′-UTR of circ_ITGA7 or SMAD4 was constructed by GenePharma. Then HCT116 and DLD1 cells were co-transfected with 50 ng pGL3 Vector and 10 ng pRL-TK Renilla together with miR-766 mimic or mimic control when cells reached 70% confluence. Cells were lysed 36 h later, and 20 µL of cell lysates were used to evaluate the luciferase activity.

### 2.10 Tumor xenografts *in vivo*

This animal study was approved by the animal care and experiment committee of Gaozhou People’s Hospital. Five-week-old BALB/c nude mice (*N* = 24) purchased from Charles River Labs (Beijing, China) were used in this study. Twenty days after injection of Lenti-circ_ITGA7 or Lenti-NC-infected HCP116 cells (2 × 10^6^/0.2 mL PBS) in mice (*N* = 6/each group), the mice were irradiated with 6 Gy once per day for the following 5 days. The tumor volume was assayed every 5 days using the formula: (length  ×  width^2^)/2. At Day 40, mice were sacrificed, the tumors were excised, and the tumor weights were recorded. The expression levels of circ_ITGA7, miR-766, and SMAD4 in tumors of mice were then examined, respectively.

### 2.11 Statistical analyses

All experiments were conducted in three independent biological replicate, and data were plotted as mean  ±  standard deviation. Group comparison was conducted using Student’s *t*-test (two-tailed) or one-way analysis of variance. The survival rates were evaluated by Kaplan–Meier method and tested by log-rank test. The relationship between two variables was evaluated using Pearson’s correlation coefficient. *P* < 0.05 indicated significantly difference.

## 3 Results

### 3.1 circ_ITGA7 expression is decreased in CRC tissues and cells

**Figure 1:**
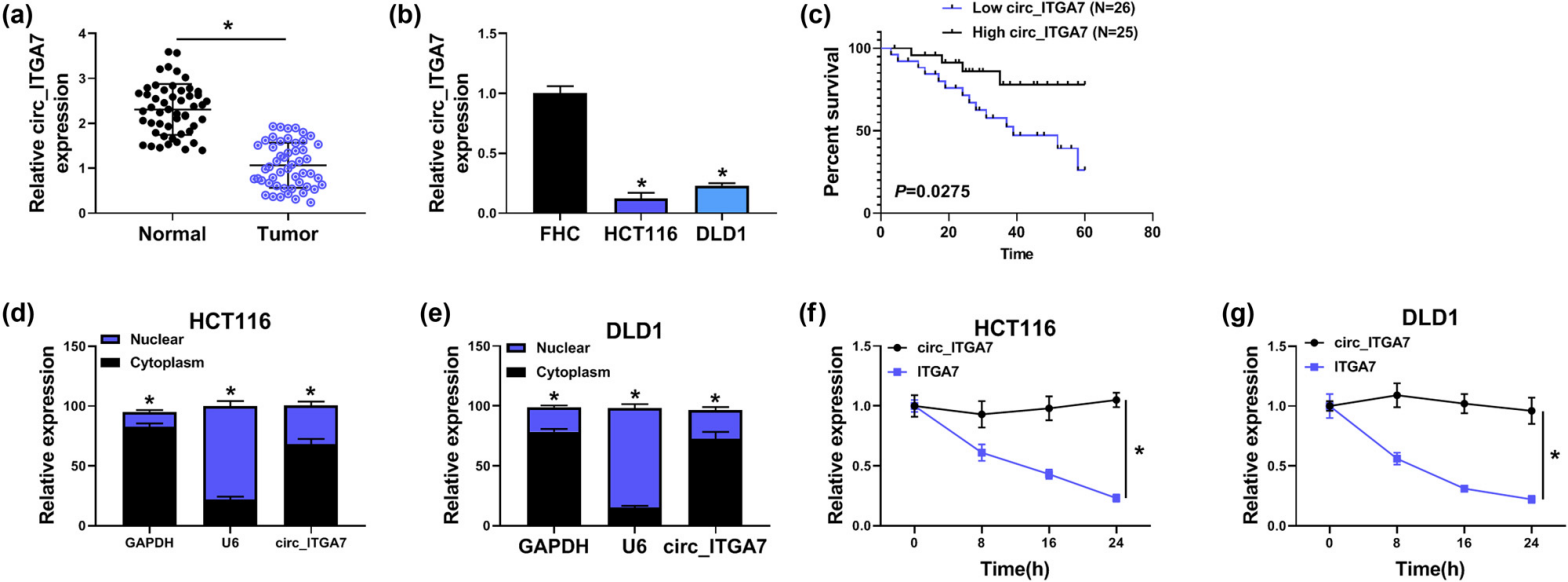
circ_ITGA7 is highly expressed in CRC tissues and cells. (a and b) Expression level of circ_ITGA7 in CRC tissues and matched normal tissues, as well as in CRC cell lines and normal FHC cells were analyzed using qRT-PCR. (c) Kaplan–Meier overall survival curve for CRC patients classified according to relative circ_ITGA7 expression level. (d and e) Cellular distribution of circ_ITGA7 was analyzed by cellular RNA fractionation assays in HCT116 and DLD1 cells. (f and g) Relative RNA levels of circ_ITGA7 and ITGA7 were measured by qRT-PCR in HCT116 and DLD1 cells after treatment with Actinomycin D at the indicated time points. *P < 0.05.

The expression profile of circ_ITGA7 was first investigated. As shown in Figure 1a and b, circ_ITGA7 expression was lower in CRC tissues and cell lines (HCT116 and DLD1) compared with the normal tissues and normal FHC cells, suggesting the involvement of circ_ITGA7 in CRC progression. Furthermore, the overall survival of CRC patients was analyzed based on the medium of circ_ITGA7 expression. Patients in high circ_ITGA7 group showed a remarkably longer overall survival than that in the low circ_ITGA7 group (Figure 1c). After that, through the use of cellular RNA fractionation, circ_ITGA7 was found to be predominately distributed in the cytoplasm of HCT116 and DLD1 cells (Figure 1d and e). Moreover, we adopted Actinomycin D to block transcription and then detected the half-life of circ_ITGA7 and ITGA7 mRNA in HCT116 and DLD1 cells. The results exhibited that circ_ITGA7 was more stable than ITGA7 mRNA (Figure 1f and g). These data demonstrated that a relatively stable cytoplasmic transcript is decreased in CRC.

### 3.2 circ_ITGA7 overexpression suppresses cell growth and enhances radiosensitivity in CRC *in vitro*

**Figure 2:**
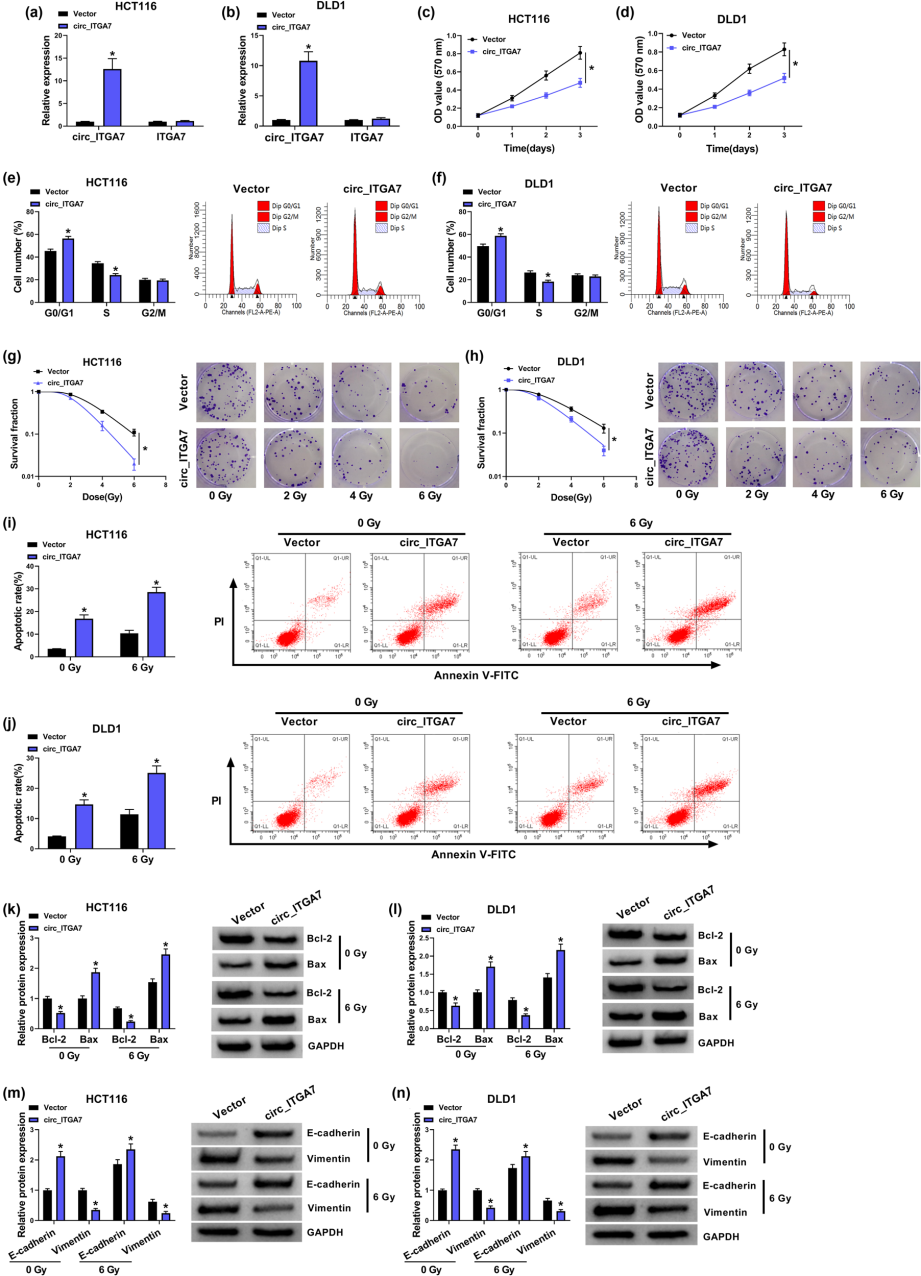
circ_ITGA7 overexpression suppresses cell growth and enhances radiosensitivity in CRC *in vitro*. (a–l) HCT116 and DLD1 cells were transfected with Vector or circ_ITGA7. (a and b) qRT-PCR analysis of circ_ITGA7 expression in HCT116 and DLD1 cells. (c and d) CCK-8 assay for the proliferation of HCT116 and DLD1 cells. (e and f) Flow cytometry for cell cycle distribution in HCT116 and DLD1 cells. (g and h) Colony formation assay for survival fraction in transfected HCT116 and DLD1 cells exposed to various doses of irradiation (0, 2, 4, or 6 Gy). (i and j) Flow cytometry for the apoptosis of transfected HCT116 and DLD1 cells exposed to 0 or 6 Gy irradiation. (k–n) Western blot analysis of Bax, Bcl-2, E-cadherin, and Vimentin protein levels in transfected HCT116 and DLD1 cells exposed to 0 or 6 Gy irradiation. *P < 0.05.

To underlie the effects of circ_ITGA7 on CRC cell growth and radiosensitivity, circ_ITGA7 overexpressing vector was designed and transfected into HCT116 and DLD1 cells. qRT-PCR analysis showed a significant elevation of circ_ITGA7 expression level in HCT116 and DLD1 cells after circ_ITGA7 transfection compared with Vector transfection (Figure 2a and b). Then CCK-8 assay suggested that circ_ITGA7 up-regulation led to a decrease of cell proliferation rate in HCT116 and DLD1 cells (Figure 2c and d). Cell cycle analysis showed that overexpression of circ_ITGA7 caused CRC cell arrest at G0/G1 phase, accompanied by the decreased percentage of cells in S phase, suggesting the inhibition of cell cycle in HCT116 and DLD1 cells (Figure 2e and f). We then explored the role of circ_ITGA7 in irradiation sensitivity of CRC cells. Transfected HCT116 and DLD1 cells were treated with different doses of irradiation (0, 2, 4, or 6 Gy). It was manifested that the transfection of circ_ITGA7 into HCT116 and DLD1 cells markedly decreased cell survival fraction with the increase of radiotherapy dose (Figure 2g and h). In addition, the irradiation treatment of 6 Gy had high-efficiency effects on the two cells. Therefore, 6 Gy dose was selected as the treatment dose. Radiosensitivity is often related to cell apoptosis, thus cell apoptosis was then analyzed. Flow cytometry suggested that circ_ITGA7 overexpression dramatically reinforced irradiation-induced apoptosis in HCT116 and DLD1 cells reflected with decreased apoptosis rate and Bcl-2 expression as well as increased Bax expression (Figure 2i–l). Besides, the action of epithelial–mesenchymal transition (EMT) in cancer drug resistance has been increasingly recognized, cells undergoing EMT exhibit a feature similar to cancer stem cells, such as an increase in drug efflux pumps and anti-apoptotic effects [14]. It has been identified that EMT can endow the cancer cells with radioresistance [15]. Then, the inhibition of Vimentin and promotion of E-cadherin mediated by irradiation were enhanced by circ_ITGA7 overexpression (Figure 2m and n), indicating that circ_ITGA7 enhanced radiosensitivity by suppressing EMT process. Taken together, circ_ITGA7 suppressed CRC cell growth and induced irradiation sensitivity *in vitro*.

### 3.3 miR-766 is a target of circ_ITGA7

**Figure 3:**
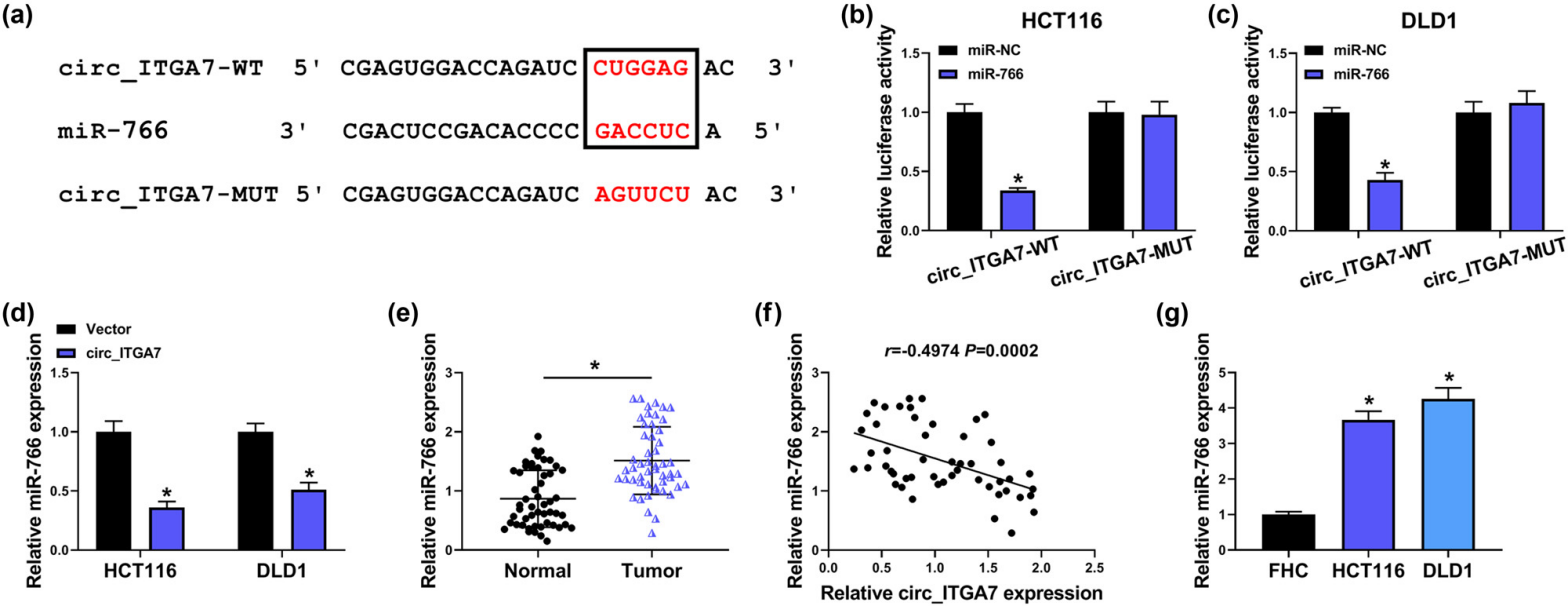
miR-766 is a target of circ_ITGA7. (a) Schematic diagram representing the predicted binding sites for miR-766 in circ_ITGA7. (b and c) Dual-luciferase activity assay for the detection of the relative luciferase activity of wild and mutated circ_ITGA7 reporter after miR-766 up-regulation in HCT116 and DLD1 cells. (d) qRT-PCR analysis of miR-766 expression in HCT116 and DLD1 cells transfected with Vector or circ_ITGA7. (e) qRT-PCR analysis of miR-766 expression in CRC tissues and matched normal tissues. (f) Pearson’s correlation coefficient analysis for the correlation between miR-766 in circ_ITGA7 expression in CRC tissues. (g) qRT-PCR analysis of miR-766 expression in CRC cell lines and normal FHC cells. *P < 0.05.

Since circ_ITGA7 was distributed predominantly in the cell cytoplasm, we hypothesized that circ_ITGA7 might act as a miRNA sponge to exert its biological functions. Through the circInteractome database (https://circinteractome.nia.nih.gov/), we identified that miR-766 might be a target of circ_ITGA7 (Figure 3a). To validate our speculation, dual-luciferase activity assay was performed. The results showed that miR-766 overexpression greatly reduced the luciferase activity in HCT116 and DLD1 cells transfected with circ_ITGA7-WT vector, while no significant effect was observed in cells transfected with circ_ITGA7-MUT vector (Figure 3b and c). Besides that, it was also found that circ_ITGA7 overexpression decreased miR-766 expression in HCT116 and DLD1 cells (Figure 3d). Therefore, we confirmed that circ_ITGA7 directly targeted miR-766 and repressed its expression. Meanwhile, miR-766 was found to be highly expressed in CRC tissues (Figure 3e), which was negatively correlated with circ_ITGA7 expression (Figure 3f). Similarly, miR-766 expression was also increased in CRC cells (Figure 3g), suggesting the potential implication in CRC progression.

### 3.4 circ_ITGA7 suppresses cell growth and enhances radiosensitivity in CRC via regulating miR-766

**Figure 4:**
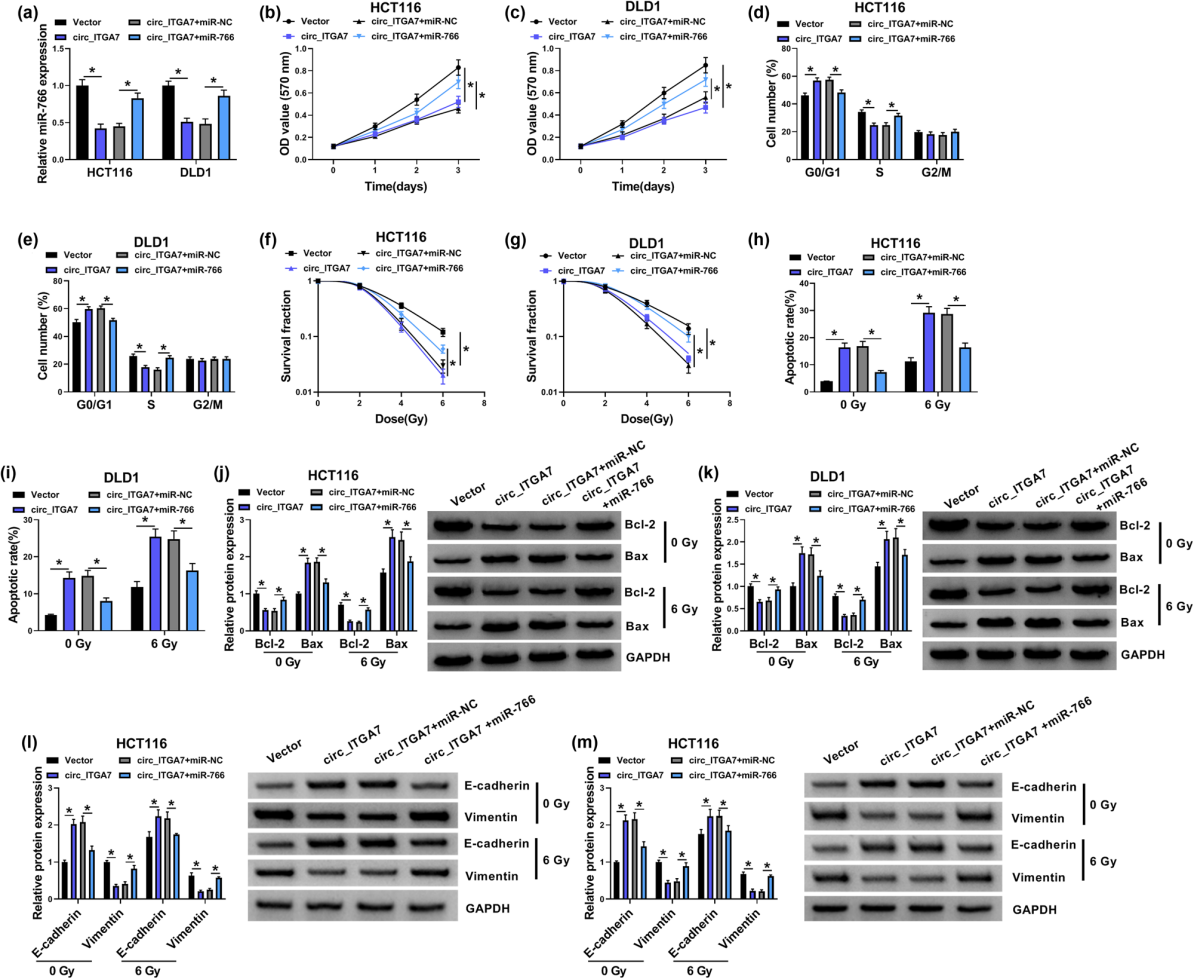
circ_ITGA7 suppresses cell growth and enhances radiosensitivity in CRC via regulating miR-766. (a–k) HCT116 and DLD1 cells were transfected with Vector, circ_ITGA7, circ_ITGA7 + miR-NC, or circ_ITGA7 + miR-766. (a) qRT-PCR analysis of miR-766 expression in HCT116 and DLD1 cells. (b and c) CCK-8 assay for the proliferation of HCT116 and DLD1 cells. (d and e) Flow cytometry for cell cycle distribution in HCT116 and DLD1 cells. (f and g) Colony formation assay for survival fraction in transfected HCT116 and DLD1 cells exposed to various doses of irradiation (0, 2, 4, or 6 Gy). (h and i) Flow cytometry for the apoptosis of transfected HCT116 and DLD1 cells exposed to 0 or 6 Gy irradiation. (j–m) Detection of Bax, Bcl-2, E-cadherin, and Vimentin protein levels in transfected HCT116 and DLD1 cells exposed to 0 or 6 Gy irradiation using western blot. *P < 0.05.

To investigate whether miR-766 was responsible for circ_ITGA7-mediated growth and radiosensitivity of CRC cells, miR-766 mimic was transfected into circ_ITGA7-increased HCT116 and DLD1 cells, as expected, miR-766 mimic rescued circ_ITGA7-induced decrease of miR-766 expression level (Figure 4a). Thereafter, the results of CCK-8 assay and flow cytometry exhibited that introduction of miR-766 mimic reversed circ_ITGA7-mediated inhibition of HCT116 and DLD1 cell growth by increasing cell proliferation (Figure 4b and c) and inducing cell cycle progression (Figure 4d and e). Colony formation assay indicated that miR-766 mimic resulted in an increase of cell survival fraction in circ_ITGA7-increased HCT116 and DLD1 cells with the increase of radiotherapy dose (Figure 4f and g). Additionally, circ_ITGA7 promoted irradiation-induced apoptosis in HCT116 and DLD1 cells accompanied by the decrease of Bcl-2 expression and the increase of Bax expression, which were abolished by miR-766 mimic (Figure 4h–k). Moreover, miR-766 overexpression reversed circ_ITGA7-caused arrest of EMT process under irradiation treatment, evidenced by the increased Vimentin and decreased E-cadherin (Figure 4l and m). Altogether, miR-766 was a functional target of circ_ITGA7 and involved in circ_ITGA7-mediated growth inhibition and radiosensitivity in CRC cells.

### 3.5 SMAD4 is a target of miR-766

**Figure 5:**
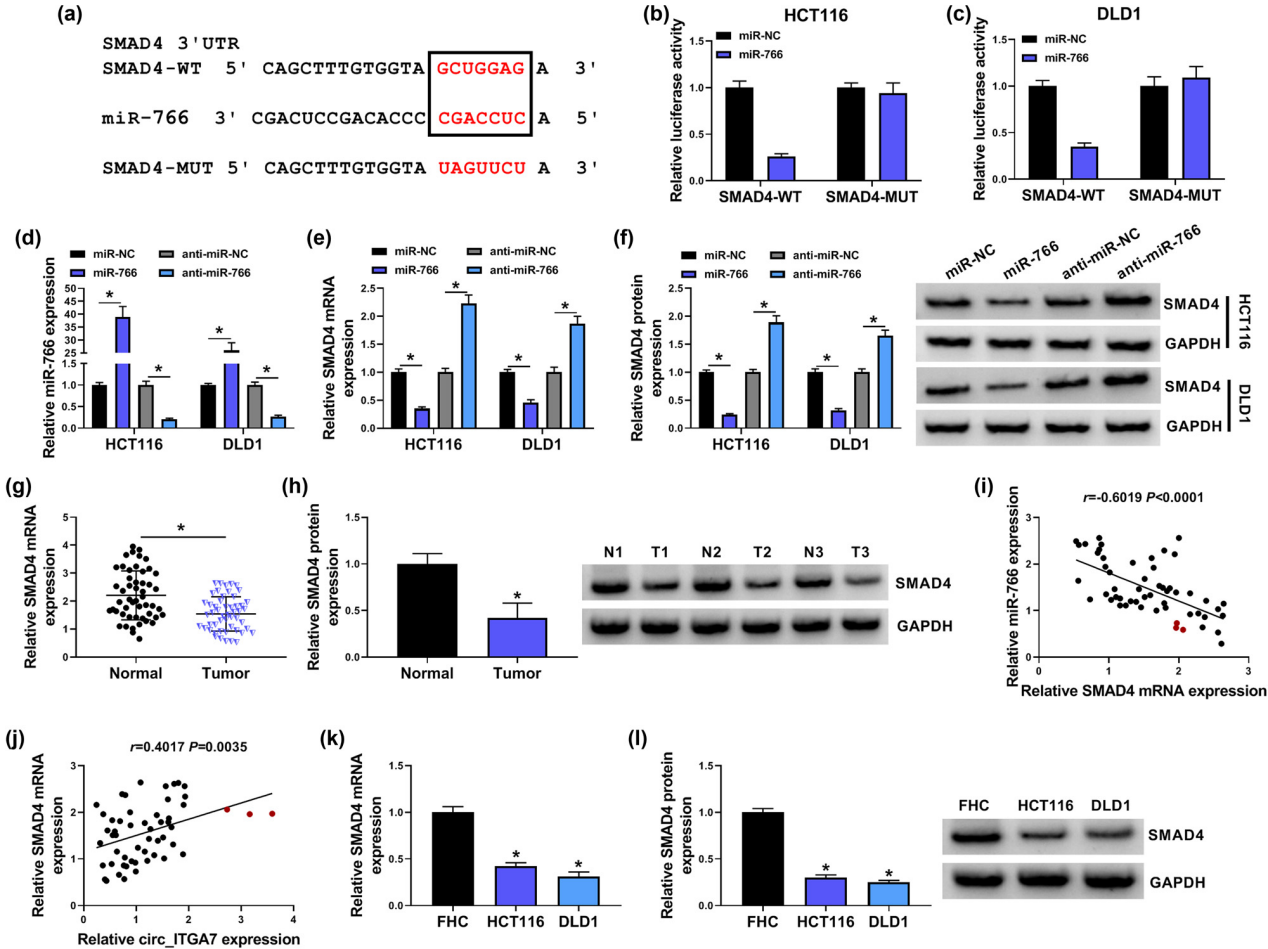
SMAD4 is a target of miR-766. (a) Schematic diagram representing the predicted binding sites for miR-766 in SMAD4. (b and c) Dual-luciferase activity assay for the detection of the relative luciferase activity of wild and mutated SMAD4 reporter after miR-766 up-regulation in HCT116 and DLD1 cells. (d–f) HCT116 and DLD1 cells were transfected with miR-766 mimic, inhibitor, or their negative control. (d) qRT-PCR analysis of miR-766 expression in HCT116 and DLD1 cells. (e and f) qRT-PCR and western blot analysis of SMAD4 levels in HCT116 and DLD1 cells. (g) qRT-PCR of SMAD4 mRNA levels in CRC tissues and matched normal tissues. (h) Western blot analysis of SMAD4 protein levels in three pairs of CRC tissues (T1–T3) and matched normal tissues (N1–N3). (i and j) Pearson’s correlation coefficient analysis for the correlation between SMAD4 and miR-766 or circ_ITGA7 expression in CRC tissues. (k and l) qRT-PCR and western blot analysis of SMAD4 levels in CRC cell lines and normal FHC cells. *P < 0.05.

Based on the prediction of miRDB database (http://www.mirdb.org/), we inferred SMAD4 transcript might be a potential target of miR-766 (Figure 5a). The results of dual-luciferase activity assay exhibited that miR-766 mimic reduced the luciferase activity of SMAD4-WT vector but not the mutated one in HCT116 and DLD1 cells (Figure 5b and c). In addition, after confirming the transfection efficiency of miR-766 mimic or inhibitor in HCT116 and DLD1 cells, it was observed that SMAD4 expression both at mRNA and protein levels was decreased in miR-766-overexpressed cells, while it was increased in miR-766-down-regulated cells (Figure 5e and f). Afterward, the expression profile of SMAD4 was determined. SMAD4 mRNA and protein levels were decreased in CRC tissues compared with the normal tissues (Figure 5g and h), which was negatively correlated with miR-766 (Figure 5i) and positively correlated with circ_ITGA7 expression at the mRNA level (Figure 5j). Also, a decreased SMAD4 expression was detected in CRC cells both at mRNA and protein levels (Figure 5k and l). Therefore, these results verified that miR-766 targeted SMAD4 and negatively regulated its expression in a targeted manner.

### 3.6 Inhibition of miR-766 suppresses cell growth and enhances radiosensitivity in CRC via SMAD4

**Figure 6:**
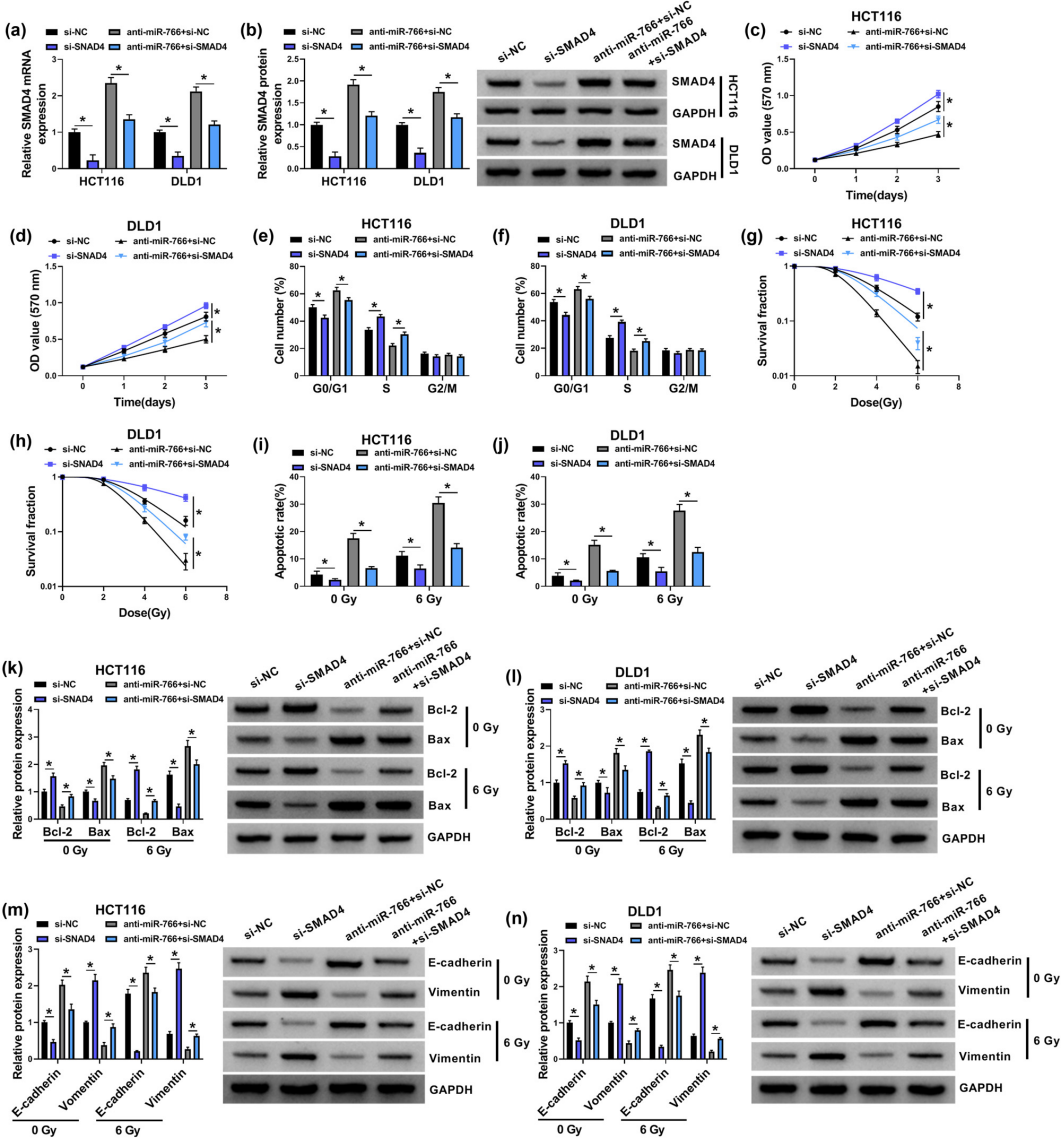
Inhibition of miR-766 suppresses cell growth and enhances radiosensitivity in CRC via SMAD4. (a–l) HCT116 and DLD1 cells were co-transfected with anti-miR-NC, anti-miR-766, anti-miR-766 + si-NC, or anti-miR-766 + si-SMAD4. (a and b) qRT-PCR and western blot analysis of SMAD4 expression in HCT116 and DLD1 cells. (c and d) CCK-8 assay for the proliferation of HCT116 and DLD1 cells. (e and f) Flow cytometry for cell cycle distribution in HCT116 and DLD1 cells. (g and h) Colony formation assay for survival fraction in transfected HCT116 and DLD1 cells exposed to various doses of irradiation (0, 2, 4, or 6 Gy). (i and j) Flow cytometry for the apoptosis of transfected HCT116 and DLD1 cells exposed to 0 or 6 Gy irradiation. (k–n) Western blot analysis of Bax, Bcl-2, E-cadherin, and Vimentin protein levels in transfected HCT116 and DLD1 cells exposed to 0 or 6 Gy irradiation. *P < 0.05.

We then elucidated whether miR-766/SMAD4 axis was engaged in CRC growth and radiosensitivity. HCT116 and DLD1 cells were co-transfected with si-NC, si-SMAD4, anti-miR-766 + si-NC, or anti-miR-766 + si-SMAD4, then we found that si-SMAD4 introduction markedly reduced SMAD4 expression, and co-transfection of anti-miR-766 + si-SMAD4 caused a decrease of the mRNA and protein level of SMAD4 in cells relative to anti-miR-766 + si-NC transfection (Figure 6a and b). The results showed that SMAD4 knockdown led to the promotion of the proliferation rate (Figure 6c and d) and cell cycle process (Figure 6e and f) in HCT116 and DLD1 cells. Besides that, with different doses of irradiation (0, 2, 4, or 6 Gy), SMAD4 knockdown markedly increased cell survival fraction (Figure 6g and h). Further, flow cytometry analysis showed that SMAD4 silencing reversed irradiation-induced apoptosis in HCT116 and DLD1 cells (Figure 6i and j) accompanied with increased Bcl-2 and decreased Bax (Figure 6k and l). In addition, western blot analysis also showed that knockdown of SMAD4 attenuated irradiation-induced EMT arrest (Figure 6m and n). Thereafter, SMAD4 might have anti-cancer effects and sensitized CRC to irradiation treatment.

Then rescue assay was conducted. miR-766 inhibitor suppressed cell proliferation (Figure 6c and d) and resulted in cell cycle arrest (Figure 6e and f) in HCT116 and DLD1 cells, while these effects were attenuated by SMAD4 knockdown (Figure 6c–f). Then transfected cells were exposed to various doses of irradiation (0, 2, 4, or 6 Gy). It was discovered that miR-766 inhibitor promoted the inhibitory effect of irradiation on HCT116 and DLD1 cell survival, evidenced by the decreased cell survival fraction, which was rescued by SMAD4 silencing (Figure 6g and h). Moreover, inhibition of miR-766 combined with irradiation elevated cell apoptosis rate in HCT116 and DLD1 cells, while co-transection of miR-766 inhibitor and SMAD4 siRNA showed decreased ratio of apoptotic HCT116 and DLD1 cells (Figure 6i–l). Conclusively, we conducted western blot analysis and showed that miR-766 inhibitor combined with irradiation suppressed EMT process, which were reversed by SMAD4 silencing (Figure 6m and n). Collectively, these data demonstrated that inhibition of miR-766 could suppress cell growth and enhance radiosensitivity in CRC in a SMAD4-dependent manner.

### 3.7 circ_ITGA7/miR-766 axis mediates SMAD4 expression

**Figure 7:**
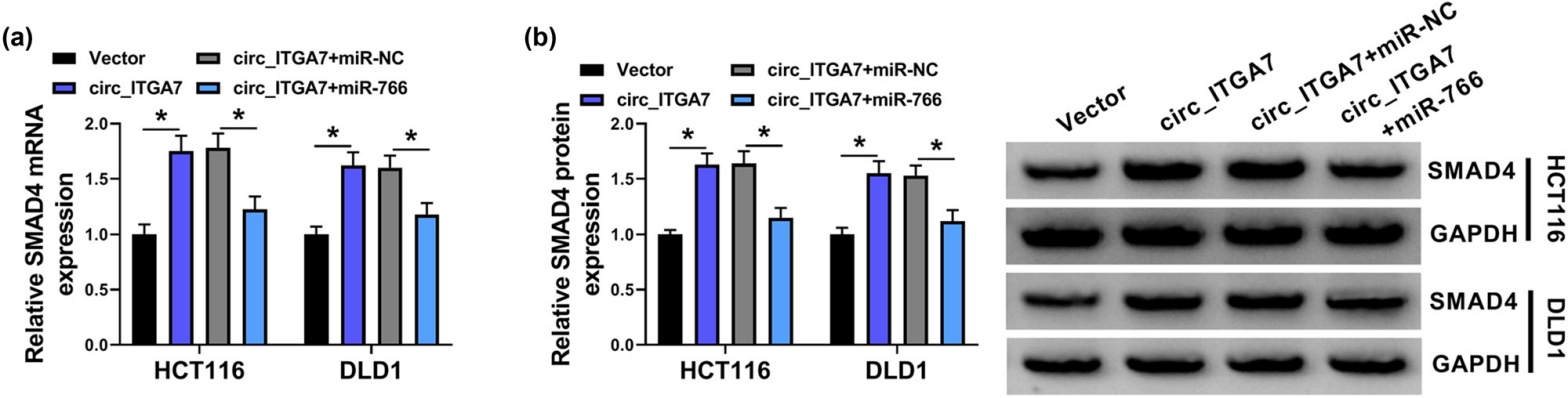
circ_ITGA7/miR-766 axis mediates SMAD4 expression. (a and b) qRT-PCR and western blot analysis of SMAD4 levels in HCT116 and DLD1 cells transfected with Vector, circ_ITGA7, circ_ITGA7 + miR-NC, or circ_ITGA7 + miR-766. *P < 0.05.

We have shown that SMAD4 was involved in the tumorigenesis of CRC, and a potent transcription target of miR-766. Thus, we validated that circ_ITGA7/miR-766 axis indeed affected the SMAD4 expression by the competing endogenous RNA hypothesis. As shown in Figure 7a and b, it was observed that circ_ITGA7 could up-regulate SMAD4 mRNA and protein expression levels in HCT116 and DLD1 cells, which were reduced by miR-766 overexpression. Therefore, we concluded that circ_ITGA7 could regulate SMAD4 expression via sponging miR-766.

### 3.8 circ_ITGA7 impedes tumor growth and enhances irradiation sensitivity *in vivo*

**Figure 8:**
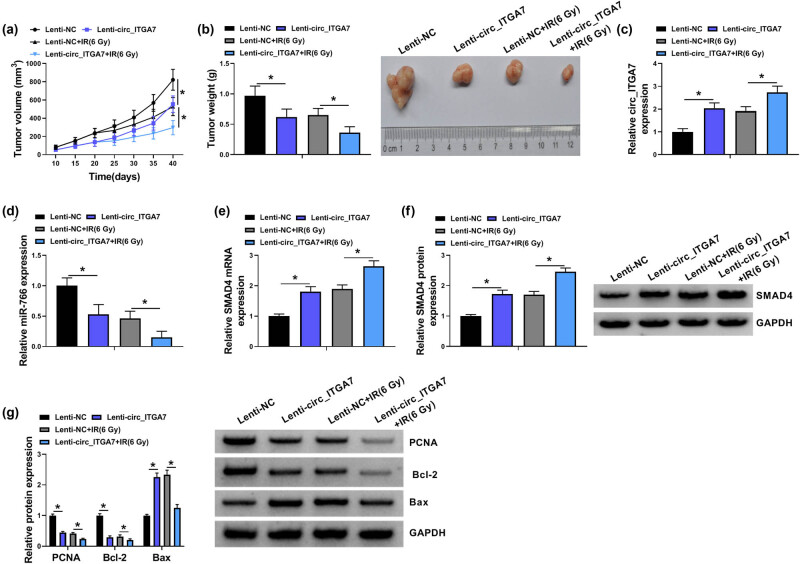
circ_ITGA7 impedes tumor growth and enhances irradiation sensitivity *in vivo.* (a) Tumor volume of each group was measured every 5 days starting 10 days after inoculation. (b) Tumor weight of each group was analyzed at Day 40 and the representative images of xenografts are shown. (c–f) qRT-PCR and western blot analysis of circ_ITGA7, miR-766, and SMAD4 expression levels in tumors isolated from mice of each group. (g) Western blot analysis of PCNA, Bcl-2, and Bax protein levels in tumors isolated from mice of each group. *P < 0.05.

We further clarified the role of circ_ITGA7 *in vivo*. A xenograft model was established by injecting Lenti-circ_ITGA7 or Lenti-NC-infected HCT116 cells into mice. Consistent with *in vitro* results, circ_ITGA7 overexpression reduced tumor volume and weight; moreover, circ_ITGA7 overexpression combined with irradiation led to a synergistic inhibition on tumor growth (Figure 8a and b). After that, molecular analysis was performed. As exhibited in Figure 8c–f, we found that circ_ITGA7 and SMAD4 expression levels were increased and miR-766 expression was decreased in circ_ITGA7-overexpressed tumor group; importantly, these effects could be enhanced by irradiation treatment. Besides that, western blot analysis also showed that circ_ITGA7 overexpression led to the decrease of PCNA, a key factor in DNA replication and cell cycle regulation, combined with Bcl-2 decrease and Bax up-regulation, two pro-apoptosis markers, in xenograft tumors, and these effects mediated by circ_ITGA7 were reinforced by irradiation treatment (Figure 8g). These data suggested *in vivo* that the up-regulation of circ_ITGA7 was sufficient to sensitize CRC xenografts to irradiation and suppressed CRC xenograft growth.

## 4 Discussion

CRC is the fourth most common cause of cancer death in the world with a high rate of incidence [16]. Preoperative RT is the primary treatment modality for CRC patients, which leads to DNA double-stranded breaks via direct DNA ionization or indirect reactive oxygen species stimulation [3]. Moreover, increasing evidence reports that neoadjuvant chemoradiotherapy benefits in controlling the local recurrence and improving the outcome of rectal cancer [17,18]. However, the occurrence of therapy resistance has been found in CRC patients which causes the failure in improving the outcomes.

Recently, it has been revealed that circRNAs have roles in regulating tumorigenesis and RT sensitization in CRC through modulating biological behaviors including cell cycle, proliferation, and apoptosis [19,20]. For instance, Bian’s team showed that hsa_circRNA_103809 impaired CRC cell migration and growth via increasing FOXO4 level through miR-532-3p [8]. Knockdown of hsa_circ_0001313 enhanced radiosensitivity by reducing cancer cell proliferation and promoting cell apoptosis through miR-338-3p [21]. Thus, targeting circRNAs may be an innovative anti-tumor approach for CRC treatment. In this study, we found that circ_ITGA7 expression was lower in CRC. Functional studies suggested the anti-cancer roles of circ_ITGA7 in restraining cell proliferation and cell cycle progression. In addition, it was also confirmed that circ_ITGA7 combined with irradiation treatment significantly reduced CRC cell survival, enhanced cell apoptosis, and arrest EMT process. Besides that, xenograft formation assay revealed that circ_ITGA7 suppressed CRC tumor growth and sensitized the CRC xenografts to irradiation *in vivo*. Besides, we also observed that radiation treatment could increase circ_ITGA7 level in CRC tumors; however, the mechanism is still unknown, and it shall be explored in future studies.

Previous studies have documented that circRNAs located in the cytoplasm can participate in gene regulation at the posttranscriptional level by acting as miRNA sponges [22,23]. This study confirmed that circ_ITGA7 was preferentially localized in the cytoplasm; therefore, the direct miRNAs interacted by circ_ITGA7 were then investigated. We confirmed that circ_ITGA7 directly targeted miR-766. Besides that, we also verified that SMAD4 was a target of miR-766; moreover, circ_ITGA7 could competitively bind with miR-766 to prevent the degradation of SMAD4. Collectively, a circ_ITGA7/miR-766/SMAD4 regulatory network was identified in CRC cells.

miR-766 is a functional miRNA, and has been demonstrated to be associated with diverse cancers. However, the action of miR-766 is complicated. It was identified to act as tumor suppressor in triple negative breast cancer [24] and papillary thyroid cancer [25], or function as onco-miR in hepatocellular carcinoma [26] and lung adenocarcinoma [27]. In CRC, Li et al. showed that miR-766 served as an oncogene to promote CRC cell growth by increasing cyclin D1 and decreasing p21 through SOX6 [28]. However, whether miR-766 was involved in therapeutic resistance remained unknown. SMDA4 is a member signal transduction protein family, phosphorylated and activated by transmembrane serine–threonine receptor kinases [29]. It is a central mediator of TGF-β signaling, which plays significant roles in cancer occurrence and progression [30]. The loss of SMAD4 was related to the recurrence, immune infiltrate, and chemoresistance in CRC [31]. Besides, loss of SMAD4 led to the poor outcome in CRC and contributed to CRC metastasis by regulating CCL15-CCR1 signaling [32]. Moreover, SMAD4 inactivation increased malignancy and chemoradiation resistance in CRC [33,34]. In the current review, we demonstrated that miR-766 promoted CRC cell growth and reduced irradiation sensitization, which was attenuated by SMAD4 knockdown. Importantly, miR-766 overexpression abolished the effects of circ_ITGA7 on CRC cell growth and RT sensitivity.

**Figure 9:**
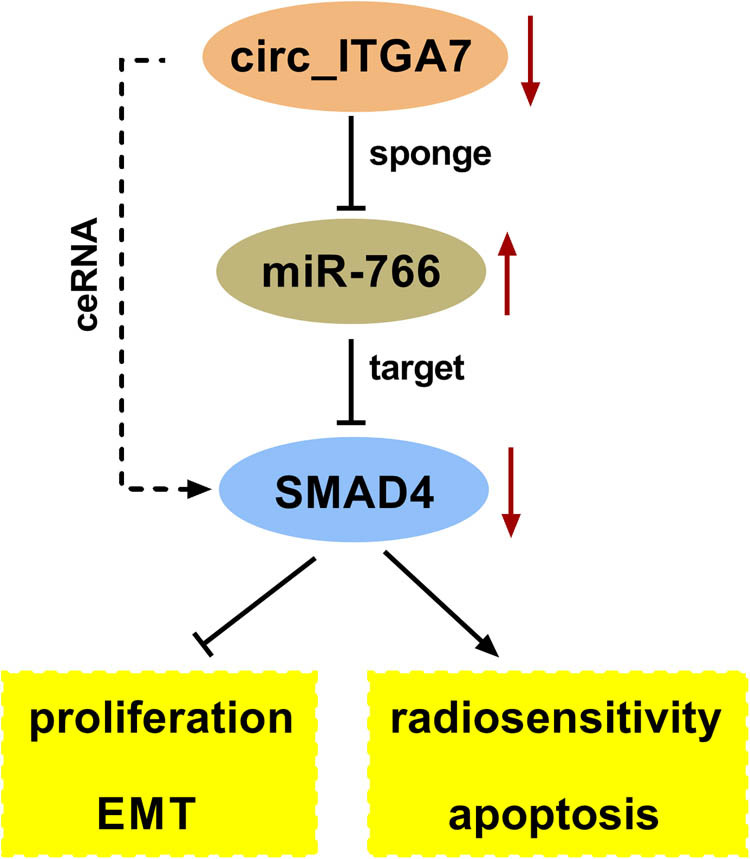
A schematic diagram illustrating how the circ_ITGA7/miR766/SMAD4 axis regulates CRC cell growth and radiosensitivity.

In conclusion, this study demonstrated that circ_ITGA7 suppressed CRC growth and enhanced RT sensitization by miR-766/SMAD4 axis (Figure 9), providing a new insight into the enhancement of irradiation therapy efficacy in CRC therapy.
